# Synergistic enhancing effect for mechanical and electrical properties of tungsten copper composites using spark plasma infiltrating sintering of copper-coated graphene

**DOI:** 10.1038/s41598-017-18114-2

**Published:** 2017-12-19

**Authors:** Wenge Chen, Longlong Dong, Jiaojiao Wang, Ying Zuo, Shuxin Ren, Yongqing Fu

**Affiliations:** 1 School of Materials Science and Engineering, Xi’an University of Technology, Shaanxi, Xi’an 710048 P. R. China; 2 Faculty of Engineering and Environment, Northumbria University, Newcastle upon Tyne, NE1 8ST UK

## Abstract

Successful applications of WCu alloys in high voltage electrical switches require their high strength and excellent conductivity. Unfortunately, the strategies for increasing their strength such as doping with fine particles and alloying often significantly decrease their conductivity. In this paper, we developed a new pathway for fabricating WCu alloys using spark plasma infiltrating sintering of copper-coated graphene (Cu@Gr) composite powders. Cu@Gr was found to partially prevent the formation of WC after sintering, and graphene was uniformly distributed on the surfaces of network Cu phases. Electrical conductivity of 38.512 M·S/m, thermal conductivity of 264 W·m^−1^·K^−1^ and microhardness of 278 HV were achieved for the sintered WCu composites doped with only 0.8 wt.% Cu@Gr powders, which showed 95.3%, 24.3%, 28% enhancement compared with those from the conventional sintering using the undoped WCu powders.

## Introduction

Tungsten copper (WCu) composites have attracted significant interest for many applications, such as electrical contacts for high voltage electrical switches, heat-sink materials for high-density integrated circuits, microelectronic blocking materials for microwave packages, and thermal transfer management in fusion power plants. This is mainly because of their combined properties of high melting points and hardness of W and excellent electrical and thermal conductivities of Cu^1^. WCu contact materials, extensively used as the key components of high-voltage circuit breakers, can efficiently transport electrical currents, however, they experience a high-temperature arc erosion over time. When the power is suddenly switched on/off, significant arcs can be easily generated and accumulated on the contact surface, thus resulting in the early failure of the material^2^. Extensive studies have been undertaken to investigate the electrical properties and arc characteristics of the WCu contact materials. Results showed that refining of grains and micro-alloying provide the WCu contact materials with higher strength and improved dispersion of arc characteristics, thus effectively inhibiting the premature ablation failure of the contacts^3–6^. However, even though the above methods have been applied, there still remains a contradictory issue to simultaneously increase both the strength and electrical conductivity of the contact materials. The refinement of grain sizes and micro-alloying improved the strength of materials and uniform dispersion of the arc energy, but the electrical properties of contact materials were decreased inevitably due to the increase of grain boundaries^7^. As there are continuous demands for the development of switch contact materials with a great capability to withstand ultrahigh voltages, it is becoming a critical issue to explore new processing techniques and new types of composites for the WCu contact materials with both superior strength and outstanding electrical properties.

Recently graphene has attracted substantial attention for its superior physical, mechanical and functional properties in the fields of metal matrix composites (MMCs)^8^. One of challenges to successfully harness graphene’s superior properties to enhance the mechanical properties of the MMCs is to uniformly disperse the graphene into metallic matrix (e.g. Al, Cu, Mg, Ni *et al*.) using powder metallurgy technologies^9–16^. Practically, surfaces of the graphene can be firstly modified using nanostructured metallic phases (such as nanoparticles of Au, Ni, etc.) which could solve the incompatability between graphene and metal matrix during fabrication, and then the surface-modified graphene can be introduced into the metal matrix using special technologies, such as laser sintering, spark plasma sintering (SPS), and electrodeposition, *et al*.^17,18^. For example, Jiang *et al*. reported an improved performance in the mechanical properties (such as tensile strength, ductility and elongation) and increased electrical conductivities for the graphene/Cu composites fabricated using spark plasma sintering (SPS) of Ni decorated graphene and Cu powders^19^. Chen *et al*. reported that Mg matrix composites with 1.2 vol. % graphene showed a 78% increase in micro-hardness^20^. In our previous work^21,22^, graphene was introduced into the WCu composites using a combined ball milling and infiltration process, and the mechanical properties (i.e, hardness) have been improved up to 121% with only 1.0 wt.% graphene being used. Simultaneously the electrical conductivity of graphene/WCu composites was increased to ~26.68 M·S/m after 0.5 wt.% of graphene was added. However, it is well-known that graphene is rather difficult to be uniformly dispersed throughout the whole WCu matrix and the wettability of graphene inside some of the metal matrix is frequently problematic^23^. Also graphene could react with W to form intermetallics such as WC, which could degrade the enhancing effects of the graphene and also decrease the electrical conductivities of the composites.

To solve the above problems, in this paper we proposed a novel method which combines both electroless plating process and SPS technology to achieve a uniform network structure of WCu composites with graphene concentrations up to 0.8 wt.%. The electroless plating was applied in order to decorate the surfaces of graphene with a uniform and controllable layer of Cu nanoparticles (hereafter we use the name of Cu@Gr). The synthesized Cu@Gr powder was then mixed with W powder, and the mixtures were sintered using the SPS.

## Results and Discussion

**Figure 1:**
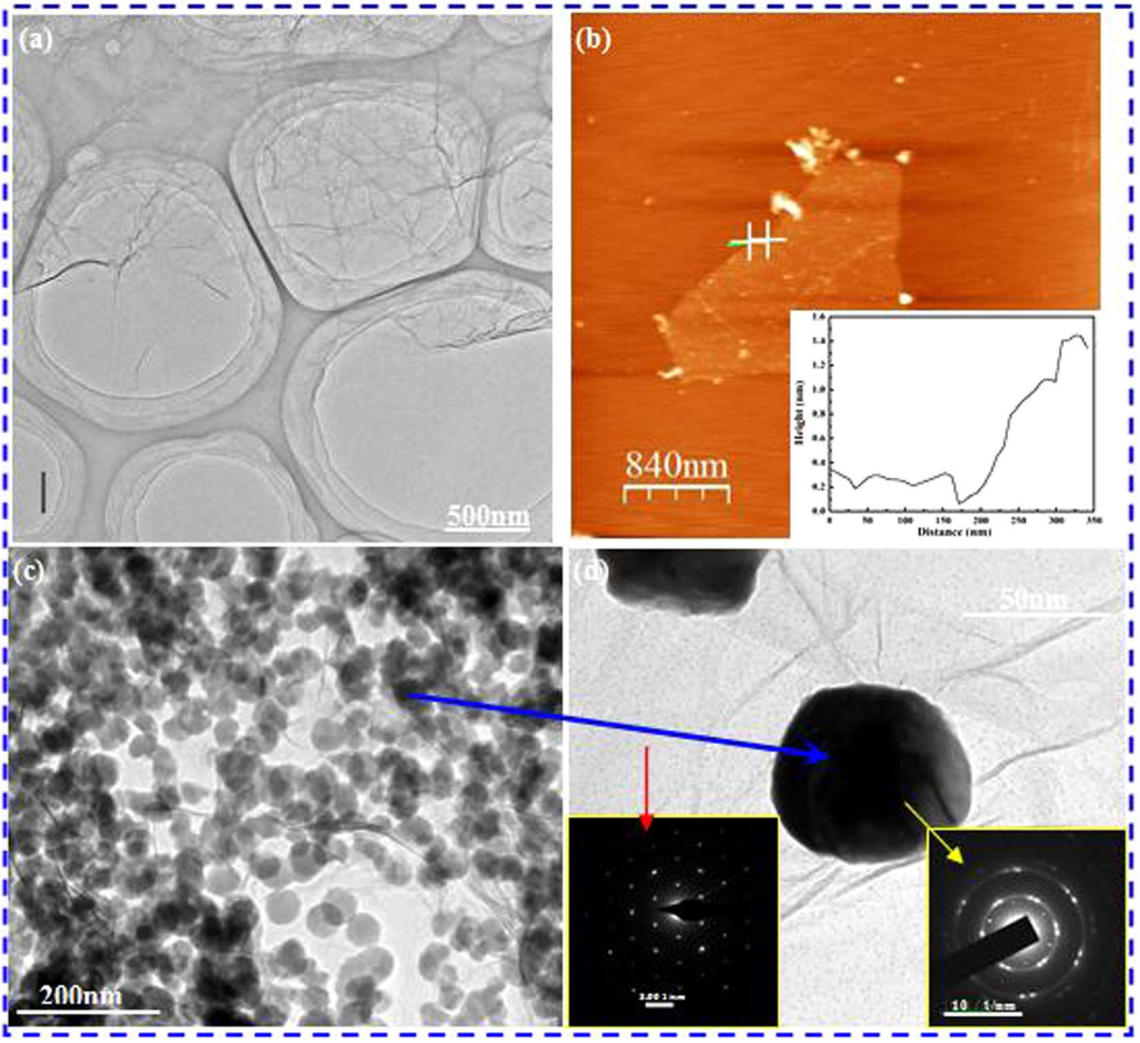
TEM images of (**a**) graphene fabricated by oxidation reduction process using a green reducing agent. (**b**) AFM image of prepared graphene in Fig. 1(a) and the inset height profile shows that the thickness of graphene is ~1.5 nm. (**c**) Cu coated graphene (Cu@Gr) prepared by electroless plating method, and (**d**) a high-magnification image of Cu@Gr powder (inset is selective area diffraction patterns of Cu and graphene, respectively).

Figure 1 shows microstructures of the graphene and Cu@Gr composite powders obtained using transmission electron microscope (TEM). Figure 1(a) shows the morphology of the prepared graphene. From Fig. 1(a), the large and thin layers of graphene with typical wrinkled structures (caused by the overlapping of graphene edges) are observed. Figure 1(b) is an atomic force microscope (AFM) image of graphene and the inset is the corresponding depth profile along the line marked in Fig. 1(b). As can be seen, the thickness of the graphene is about 1.5 nm, indicating that there are not multilayered graphene. After the electroless plating process, graphene is uniformly mixed with Cu particles with an average diameter of ~50 nm (Fig. 1(c)). In Fig. 1(d), a single Cu particle can be observed to attach well on the surface of the graphene. Diffraction patterns show clearly the structures of the graphene (as denoted by the red arrow) and coated Cu particle (as denoted by the yellow arrow).

**Figure 2:**
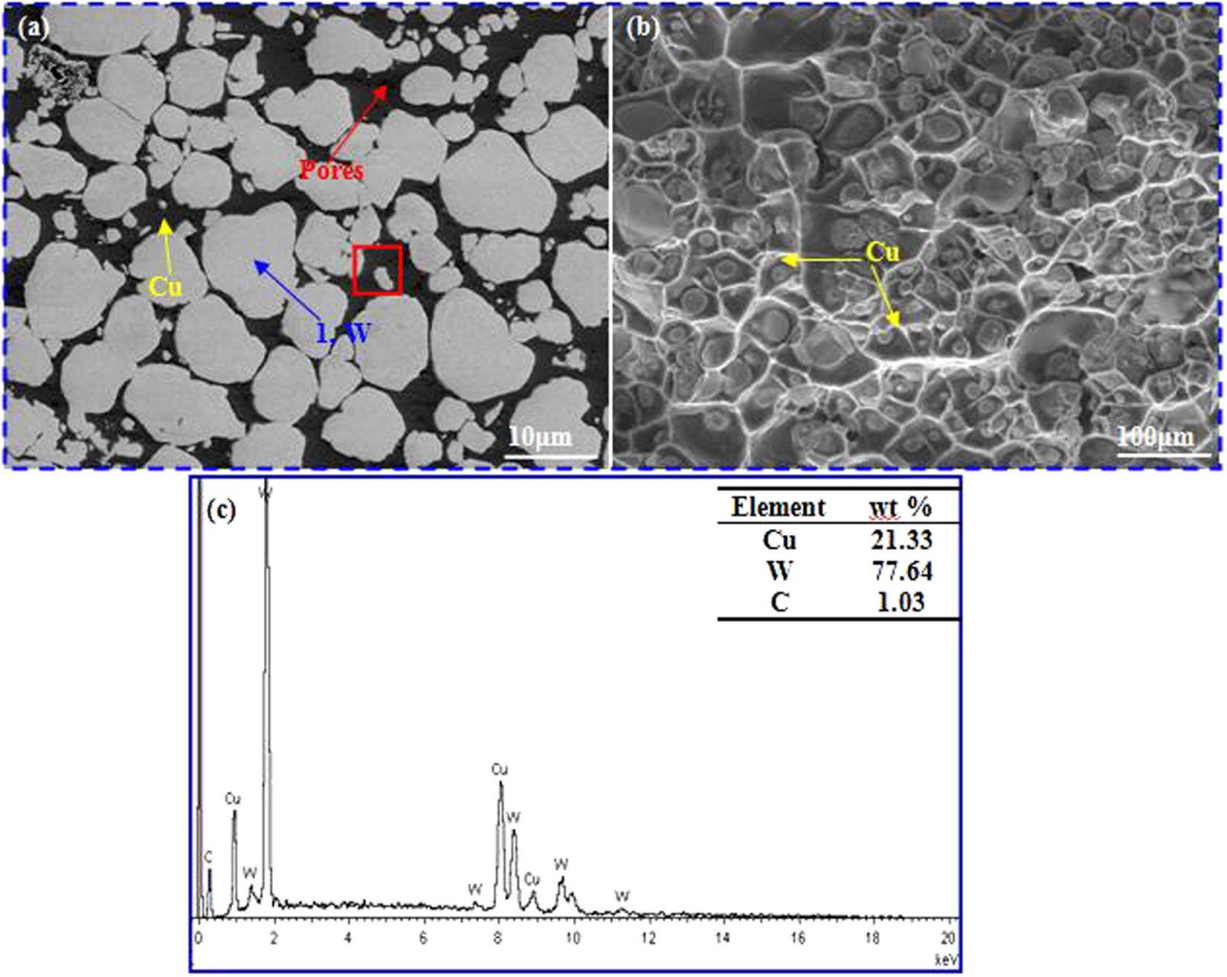
SEM images showing microstructures of Cu@Gr/WCu composites. (**a**) polished and etched cross-section; (**b**) fracture surfaces, and (**c**) EDS result of red rectangle in Fig. 2(a).

Figure 2 presents scanning electron microscope (SEM) images of the synthesized Cu@Gr/WCu composites, in which Fig. 1(a) is their cross-section morphology and Fig. 1(b) is their fracture surface. In Fig. 2(a), W-phases with bright colors (pointed by arrow 1) are distributed homogeneously inside the dark background of Cu phase. Detailed studies show that there are pores in the Cu phases due to their expansion and contraction during and after SPS process. Figure 2(b) shows a clear network distribution of Cu on the fracture surface. The presence of graphene inside the Cu phase of the composites can be identified from the energy dispersive spectroscope (EDS) analysis as shown in Fig. 2(c), and carbon element has a content of 1.03 wt.%, as shown in the inset of Fig. 2(c).

**Figure 3:**
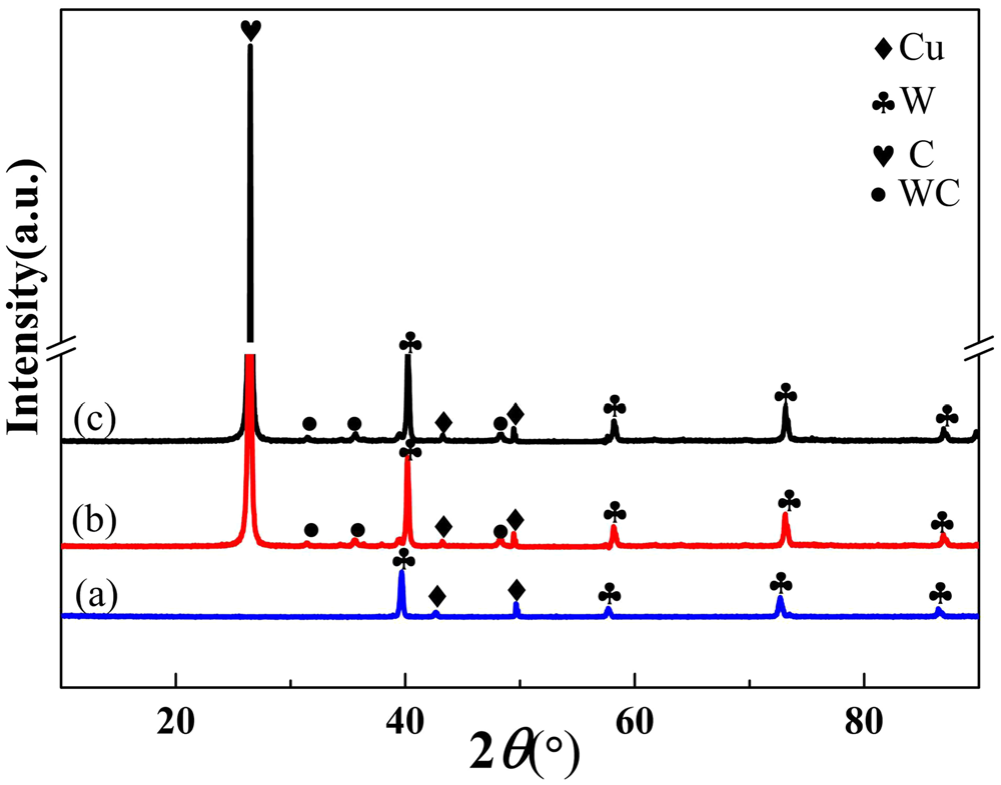
XRD patterns of (**a**) WCu composites, (**b**) doped with 0.8 wt.% graphene, and (**c**) doped with 0.8wt.% Cu@Gr.

Figure 3 shows X-ray diffraction (XRD) results of the WCu composites fabricated using the composite powders with graphene contents of 0 and 0.8 wt.%, respectively. In Fig. 3(a), the sample without adding graphene mainly shows W and Cu diffraction peaks at 2θ values of ~40.264 (110), ~58.274 (200), ~73.195 (211), and ~43.297 (111), ~50.433 (200). As shown in Fig. 3(b)~(c), it is notable that an intensive peak at 2θ = 26.1° was observed in the XRD result of the 0.8 wt.% graphene doped WCu composites. Also, minor WC phases can be identified, i.e., the weak peaks emerged at 2θ equal to 31.511 (001), 35.641 (100), 48.296 (101), respectively. By adding 0.8 wt.% of Cu@Gr in the WCu composites, the intensity of carbon diffraction peak increases significantly, as can be compared from Fig. 3(b and c).

The carbon atoms of graphene diffuse onto the surfaces of the W-W skeletons at high temperatures. They could diffuse easily into the octahedral spacings of the W atoms due to the differences of their atom diameters (i.e., d_W_ = 0.274 nm, d_C_ = 0.154 nm). The chemical reactions between W particle and graphene can be expressed using the following equations:

1 $${\rm{2W}}+{\rm{C}}\to {{\rm{W}}}_{2}{\rm{C}}\quad {\rm{\Delta }}{\rm{G}}=-30540-2.34{\rm{TJ}}/{\rm{mol}}$$

2 $${{\rm{W}}}_{2}{\rm{C}}+{\rm{C}}\to 2{\rm{WC}}\quad {\rm{\Delta }}{\rm{G}}=-16988-1.7TJ/{\rm{mol}}$$

3 $${\rm{W}}+{\rm{C}}\to {\rm{WC}}\quad {\rm{\Delta }}{\rm{G}}=-{\rm{42260}}-4.98{\rm{TJ}}/{\rm{mol}}$$

According to W-C equilibrium phase diagram^24^, Gibbs free energy values (ΔG) of Eqs (1)~(3) are negative at the temperatures ranging from 500 °C to 1500 °C, which indicates that these are the favorite conditions for the thermodynamic reactions. The value of ΔG of Eq. (3) is the lowest one when the sintering temperatures are between 1280 °C and 1300 °C, therefore, theoretically, reaction in Eq. (3) is the most likely one to occur during the same sintering conditions. However, the reaction process between W and C is also controlled by the diffusion of C atoms toward the W atom. Hence, the metastable W_2_C is easily generated when the chemical reaction occurs during SPS process. Then the activities and concentrations of the carbon atoms will be increased, leading to the transformation of metastable W_2_C into a more stable WC phase. In this work, an intensive peak of carbon can be observed as shown in Fig. 3, indicating that a large amount of carbon in the Cu@Gr was not reacted with the W particles. The remained graphene in the Cu@Gr phase will surely improve the properties of composites. Cu particles, attached closely with the graphene, also play a significant role in preventing the direct contacts between graphene and W particles, therefore, the chemical reactions between W and graphene are prevented. Otherwise, the W skeleton will react easily with C atoms at a high sintered temperature if there is a direct contact of W and graphene.

**Figure 4:**
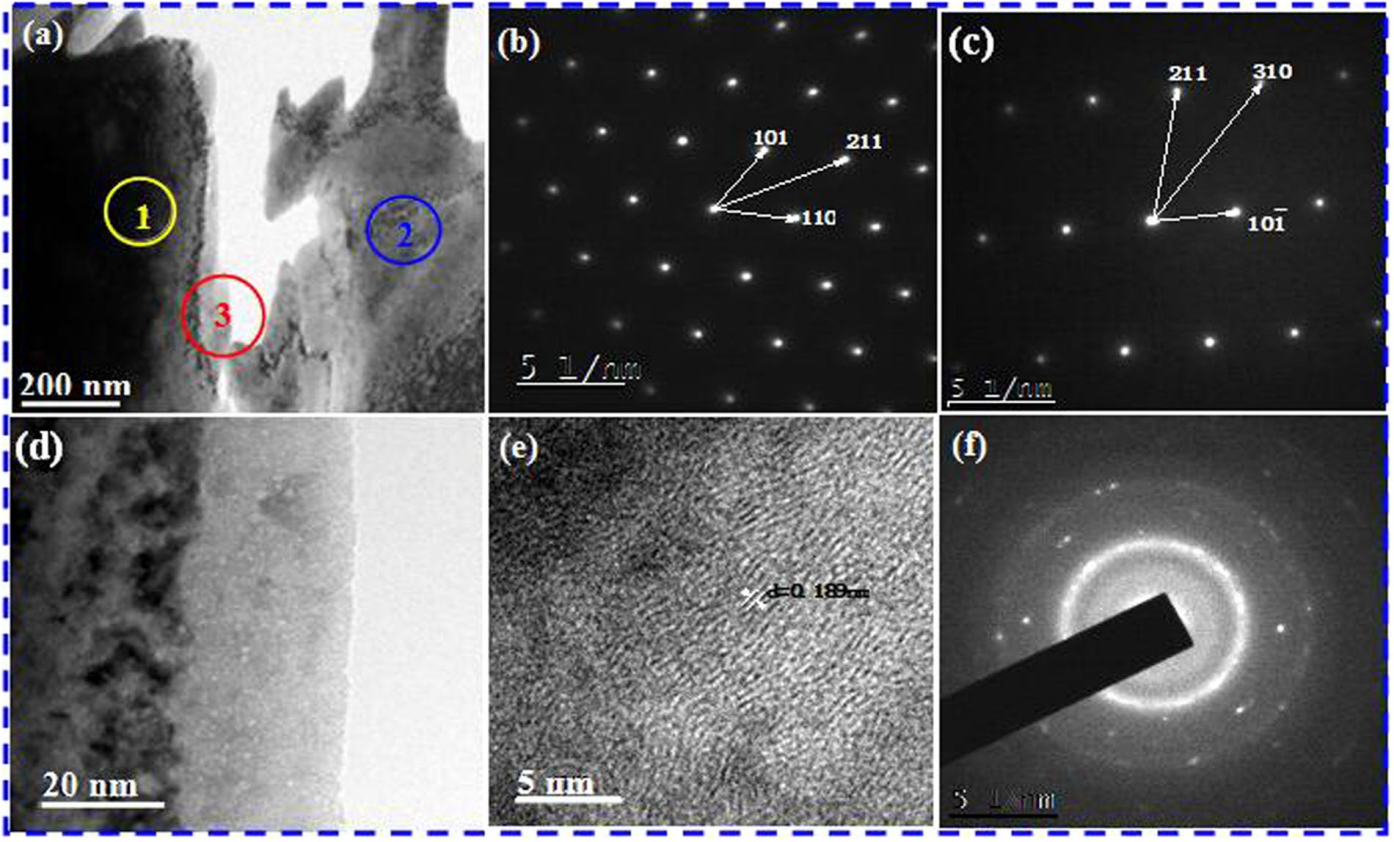
TEM images of Cu@Gr/WCu composites doped with 0.8 wt.% Cu@Gr after SPS sintering. (**a**) 0.8 wt.% Cu@Gr/WCu composites. (**b**) Electron diffraction pattern and calibration of 1 in Fig. 4(a). (**c**) Electron diffraction pattern and calibration of 2 in Fig. 4(a). (**d**) High magnification of marked 3 zone in Fig. 4(a). (**e**) High-magnification images of marked 4 zone in Fig. 4(d). (**f**) Electron diffraction pattern and calibration of marked 4 zone in Fig. 4(d).

Figure 4 shows TEM images of the WCu composites doped with 0.8 wt.% Cu@Gr. In Fig. 4(a), the existence of W phases (indicated by circle 1) and Cu phases (indicated by circles 2 and 3) can be confirmed by patterns of selected area electron diffractions (SAED) shown in Fig. 4(b) and (c). Figure 4(d) shows an enlarged morphology of circle 3 in Fig. 4(a), indicating that there are many particulate structures. The high-resolution TEM (HRTEM) image of the circle 4 in Fig. 4(d) is shown in Fig. 4(e) and its corresponding SAED result is shown in Fig. 4(f). Clearly results show that this phase is graphene because the diffraction rings are consistent with those interplanar distances of graphene^25^. The lattice fringes of Cu can be observed in the HRTEM image as shown in Fig. 4(e), and the lattice constant was calculated to be ~0.189 nm corresponding to the interplanar spacing of Cu (200). This clearly demonstrates the well-defined crystallinity of the Cu nanoparticles in the Cu@Gr particles. Therefore, we can confirm that the structure of the Cu@Gr powders was not apparently modified after the SPS process used in this work. There are no detectable new phases, except W, Cu and graphene phases, which is consistent with the XRD results shown in Fig. 3.

Table 1 lists a comparison of the measured physical, thermal and mechanical properties from the composites in this study and those from literature. Electrical conductivity of 38.512 M·S/m, thermal conductivity of 264 W·m^−1^·K^−1^ and microhardness of 278HV were achieved in the WCu composites doped with 0.8 wt.% Cu@Gr, which are 95.3%, 24.3%, 28% enhancement compared with those sintered using the undoped WCu composites^26^. With the help from discharge fields and the applied pressure during the SPS, the rapid migration of materials caused by flows of the liquid Cu results in an efficient filtration of Cu, which is then uniformly distributed inside the W skeleton. This will enhance the densification of the composites and strengthen the interfacial bonding between Cu and W. Also, there will not be any significant evolution of microstructures or increase of grain sizes during or after sintering due to the short process time of the SPS process. Thirdly, formation of tungsten carbides, produced by the mild reactions between W and graphene (see Fig. 3), plays an activated sintering role, i.e., accelerating the migration of atoms inside the composites, and thus enhancing the formation and growth of sintering necks.

**Table 1:** Comparison of physical and mechanical properties of W80Cu20 composites with those from literature.

Materials	Relative density (%)	Electrical conductivity (M·S/m)	Thermal conductivity (W·m^−1^·K^−1^)	Mechanical properties	References
W80Cu20	96.3	19.72	200	220^a^	^26^
W80Cu20-0.8wt.%Cu@Gr	99.1	38.512	264	278^a^	Present work
W80Cu20-0.4wt.%CNTs	97.2	—	228.32	1440.6^b^	^31^
W80Cu20-0.8wt.% TiN@SiC_f_	98.5	—	235	1200^b^	^32^
W70Cu30-1wt.% La_2_O_3_	92	26.68	—	940^b^	^33^

^a^Hardness (HV) value. ^b^Transverse rupture strength (TRS, MPa) value.

The important factors for densification by both solid state and liquid phase mechanisms are the two terms of solubility and diffusivity. For a conventional liquid phase sintering, the diffusivity of the base metal in the liquid is much larger than that along the grain boundary. For the case of WCu, the concentration of W in the liquid Cu is very small. As mentioned above, tungsten easily reacts with graphene, which can enhance activated sintering behavior^27^. The grain boundary diffusivity is substantially increased due to the presence of the activator (i.e. graphene) and enhanced volume (or mass) transport of W.

In addition, formation of a continuous Cu network, achievement of a high relative density of composites and successful integration of graphene into the WCu composites are the other key reasons for the good thermal and electrical properties of WCu composites obtained in this work. These microstructures will form the electron transportation channels during the measurement.

**Figure 5:**
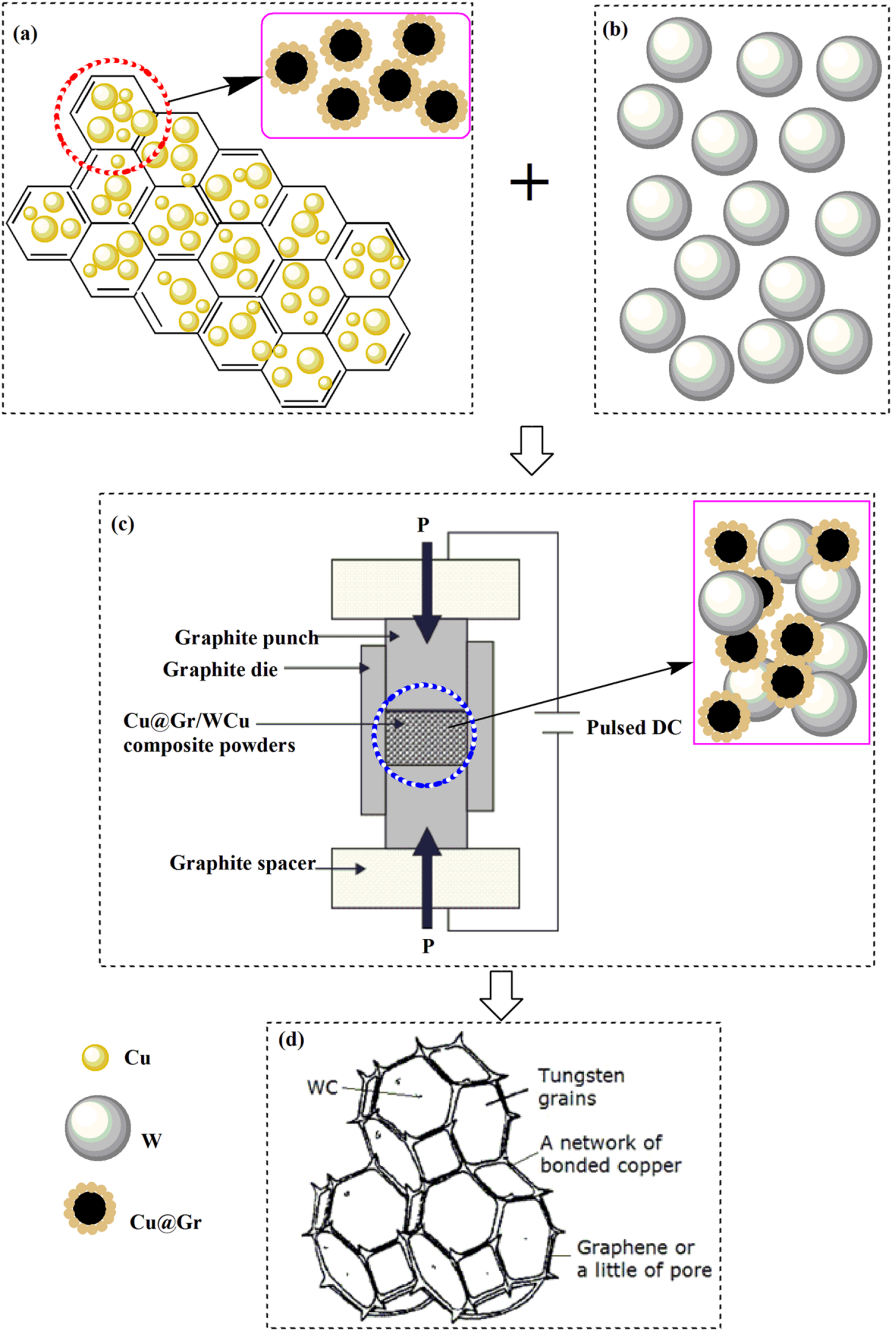
Diagram illustrating the proposed mechanism of coated powders during spark plasma infiltrating sintering.

Figure 5 illustrates the formation mechanisms of the sintered Cu@Gr/WCu composites during the SPS process. For the Cu@Gr/WCu composites, there are three key mechanisms for the changes in the properties.

1. Cu coated graphene, similar to Cu wrapped carbon layer, will hinder the chemical reaction of carbon and W, and further avoid the introduction of the impurities, as shown in Fig. 5(a). As is well known, carbon and Cu have no mutual solubility and chemical reactions. As a result, the Cu@Gr/WCu composites can obtain excellent mechanical and electrical properties because graphene and W still keep their intrinsic structures and properties.
2. In Fig. 5(b) and (c), since it is known that current takes the shortest path during the electrical measurement, in SPS, the current follows conduction path at the particle surfaces, therefore, heat is primarily concentrated on the surfaces of particles (W and Cu@Gr) during sintering. The DC pulsing cycles preserve the charge accumulated at the particle surfaces, thereby generating spark discharges and joule heat among particles. The DC current causes rapid and uniform distribution of the heat throughout the powder compact, enhancing homogeneity of microstructure and uniformity of density. The Joule’s heating and applied pressure are responsible for the enhanced diffusion and mass transport during sintering. Finally, the Cu@Gr particles can form a network type and effectively fill the gaps among the W particles. The significant grain growth could be prevented due to the shorter holding time and applying of the external pressure during the SPS^28^. All these cause the necks to be gradually developed and the grain size to be increased, thus resulting in a rapid densification sintering.
3. As illustrated in Fig. 5(d), a small amount of WC formed at contact surfaces of the W particles could inhibit the growth of W grains since the WC phases with an excellent chemical stability at high temperatures are pinned at the boundaries of W particles. Therefore, they can suppress the excessive growth and formation of sintering necks of W particles during the process of activated sintering^27^. Hence, the sintered W skeletons with WC have high porosities with interconnecting channels. This type of structure is favorable for the molten Cu to easily flow into the W skeleton structures due to the capillary action during the infiltration process. This will result in the formation of the continuous Cu phase, thus improve the electric conductivity and mechanical properties of the WCu composites (as listed in Table 1).

## Conclusions

In summary, the Cu@Gr/WCu composites were successfully fabricated using an electroless plating process and SPS process at 30 MPa and 1280 °C for 10 min. Graphene was uniformly distributed onto the surfaces of network Cu binder phase. Cu@Gr can partially prevent the formation of WC. The electrical conductivity of 38.512 M·S/m, thermal conductivity of 264 W·m^−1^·K^−1^ and microhardness of 278 HV were achieved in the WCu composites doped with 0.8 wt.% Cu@Gr, which is 95.3%, 24.3%, 28% enhancement over the undoped WCu composites. Our future work will be focused on the optimization of the processing parameters and investigation of interfacial reaction for the Cu@Gr/WCu composites, with the aim to clarify the effects of processing temperature/time on the interfacial reaction. Effect of Cu@Gr additives on electrical arc breakdown properties of WCu composites will also be investigated.

## Experimental Methods

In our proposed method, there are four key steps to fabricate Cu-coated graphene/WCu (Cu@Gr/WCu) composites based on the powder metallurgy route:

1. Graphene was prepared at the ambient temperature by oxidation reduction process of graphite using a green reducing agent (thiourea dioxide). Details of the preparation procedure can be found in our previous paper^29^.
2. Composite powders of Cu-coated graphene (Cu@Gr) were synthesized using an electroless plating method^30^. Commercially available Cu powders (99.7% purity and particle size <53 nm) and W powders (purity ≥99.9% and average particle size of 5~7μm,) were bought from Sinopharm Chemical Reagent Co., Ltd., China.
3. Powders with given compositions (i.e., 0.8 wt.% of Cu@Gr composite powders, 80 wt.% of W powders, and 20 wt.% Cu powders) were ball-milled for 24 hrs using agate balls of diameters of 2 mm in a glass container. Extra amounts of Cu powders (0.2 times of theoretical Cu content in W80Cu20 alloys) in the pre-mixed powder were added to compensate the loss of Cu during sintering. Ar gas was used during the ball milling to reduce the oxidation of the Cu powder. After ball-milling, the powders were sieved through a 20 mesh sieve to remove the agate balls. The milled powders were then compacted into cylinders (with a diameter of 11 mm and a length of 4 mm) under a pressure of 600 MPa. This resulted in a green compact density of ~13 g/cm^3^, which is ~ 85% of the theoretical density of W80Cu20 alloys.
4. Sintering of powders was performed using the SPS in Ar at temperatures ranging from 1280 °C to 1350 °C. The heating rate was 10 °C/min and the hold time at the sintering temperature was 10 min. Temperature variations were precisely controlled to be within ±5 °C. The pressure applied to the sample was 30 MPa during the SPS process.

Densities of sintered samples were measured using the Archimedes’ water immersion method according to ASTM Standard B328. Samples were sectioned and polished for Vickers micro-hardness measurements, which were performed by applying a 500 g load for 10 s. Four measurements of the micro-hardness were taken at random locations throughout the sample, and the average reading was obtained. No cracks were observed around the indentation marks. Microstructural and structure analysis of the samples were performed using both SEM (JSM-6700), along with EDS, TEM using Cu grid (TEM, JEM-3010), and AFM (E-Sweep). Crystalline structures of the sintered composites were analyzed using XRD (XRD-7000S). Thermal diffusivity (α) of the Cu@Gr/WCu composites was measured using LFA 427 Nanoflash (NETZSCH, Germany) according to ASTM Standard E1461 and an average reading from three set of data was obtained. Thermal conductivity (λ) was determined using the equation (4):

4 $${\rm{\lambda }}={\rm{\alpha }}\times {{\rm{C}}}_{{\rm{p}}}\times {\rm{\rho }},$$

in which α is thermal diffusivity, C_p_ is specific heat (and ρ is density of the obtained WCu composites. Electrical conductivity of specimen was measured using a D60K Conductivity Tester.
